# A Rare Case of Broad Ligament Hematoma Following Vaginal Delivery

**DOI:** 10.7759/cureus.62205

**Published:** 2024-06-11

**Authors:** Manju Mathesan, Nidhi Sharma

**Affiliations:** 1 Obstetrics and Gynaecology, Saveetha Institute of Medical and Technical Sciences, Chennai, IND

**Keywords:** hematoma, broad ligament hematoma, postpartum hemorrhage, atypical presentations, broad ligament, normal vaginal delivery

## Abstract

This case report explores a rare complication of broad ligament hematoma post-vaginal delivery, emphasizing the importance of prompt intervention in cases of postpartum hemorrhage with atypical presentations. A 22-year-old primigravida, at 39 weeks with hypothyroidism, presented with intermittent abdominal pain and normal fetal movements. After a normal vaginal delivery with a right mediolateral episiotomy, she developed intense perineal pain and hypotension due to a broad ligament hematoma. The surgical intervention included the evacuation of the hematoma, laparotomy, and internal iliac artery ligation. The postoperative care involved treatment for a methicillin-resistant *Staphylococcus aureus* (MRSA) infection, and the patient received blood transfusions. The follow-up showed complete wound healing and an uneventful postnatal period, with the patient resuming normal activities after three weeks. Comparison with similar cases in the literature highlighted various etiologies and clinical presentations of broad ligament hematoma, ranging from broad ligament pregnancy to uterine perforation. Timely surgical exploration, hematoma evacuation, and arterial ligation were essential in preventing adverse maternal outcomes, underscoring the importance of multidisciplinary collaboration and vigilant postoperative monitoring. The report emphasizes the need for a high index of suspicion and prompt intervention to ensure optimal recovery and minimize complications in cases of broad ligament hematoma following vaginal delivery.

## Introduction

Vaginal delivery, while common in obstetric practice, can occasionally lead to unforeseen complications. The typical risks associated with vaginal delivery include postpartum hemorrhage (PPH), perineal trauma and tears, infections, retained placenta, uterine inversion, shoulder dystocia, amniotic fluid embolism (AFE), pelvic organ prolapse, thromboembolic disorders, and psychological complications [1]. One such rare complication is a broad ligament hematoma, often resulting from trauma to the vasculature during childbirth. This case report explores the presentation, diagnosis, and management of a broad ligament hematoma following a routine vaginal delivery, highlighting the challenges and considerations in its clinical management [2].

Broad ligament hematoma is a rare but significant complication in obstetric practice, typically arising during or after childbirth. This condition involves the accumulation of blood within the broad ligament, a double layer of peritoneum that extends from the sides of the uterus to the pelvic walls, supporting the uterus, fallopian tubes, and ovaries. The broad ligament plays a critical role in maintaining the position of the reproductive organs, and hematoma formation in this area can have profound clinical implications. The occurrence of a broad ligament hematoma is often associated with trauma to the pelvic vasculature, which can happen during vaginal delivery, cesarean sections, or other pelvic surgeries. For instance, a study by Oikawa et al. [1] highlighted a case of broad ligament hematoma in a broodmare due to uterine artery rupture postpartum. Another case discussed by Luke et al. [2] involved a concealed uterine rupture leading to a hematoma within the broad ligament, underscoring the variety of causes and presentations of this condition. From a clinical perspective, patients with broad ligament hematoma typically present with intense pelvic or lower abdominal pain, hemodynamic instability (such as hypotension and tachycardia), and a palpable pelvic mass. These symptoms necessitate prompt and accurate diagnosis, which can be achieved through clinical examination, imaging modalities such as ultrasound or CT scans, and laboratory tests to evaluate the extent of blood loss.

The management of broad ligament hematoma depends on the size of the hematoma and the patient's hemodynamic status. Smaller, stable hematomas might be managed conservatively with close monitoring, fluid resuscitation, and analgesia. In contrast, larger or expanding hematomas, or those causing significant hemodynamic instability, often require surgical intervention. This can involve hematoma evacuation and ligation of the bleeding vessels. In severe cases, interventional radiological techniques such as embolization or surgical procedures such as internal iliac artery ligation may be necessary. Sabban et al. [3] reported a case managed by embolization due to PPH. The significance of recognizing and managing broad ligament hematoma in obstetric practice cannot be overstated. If left untreated, this condition can lead to severe complications, including hypovolemic shock, infection, and long-term reproductive issues. Postoperative care is crucial to monitor for potential complications, such as the risk of infection (with methicillin-resistant *Staphylococcus aureus* (MRSA) infection being a noted risk), as highlighted by the case involving MRSA infection post-surgery. In summary, broad ligament hematoma is a rare but potentially life-threatening condition that requires a high index of suspicion, timely diagnosis, and appropriate management to ensure favorable outcomes. Multidisciplinary care, including obstetricians, surgeons, and interventional radiologists, is often essential to effectively address this complex clinical scenario.

A pelvic hematoma, characterized by the accumulation of blood above the levator ani muscle, is termed a broad ligament or supralevator hematoma [3]. The occurrence of a broad ligament hematoma following a normal vaginal delivery is exceedingly rare. It is noteworthy that a substantial amount of hematoma can accumulate between the folds of the broad ligament, extending to the natural cleavage lines of the connective tissue, before manifesting as a shock [4]. Therefore, a high index of suspicion is imperative in patients presenting with signs of PPH, particularly in cases where minimal or no vaginal bleeding is observed during delivery, immediately after delivery, or later in the puerperium phase [5].

Maintaining a high index of suspicion for PPH is crucial, particularly in cases with subtle or delayed symptoms. PPH is one of the leading causes of maternal morbidity and mortality worldwide, and its timely diagnosis and management are vital for preventing severe outcomes. In cases of broad ligament hematoma, symptoms may not be immediately apparent. As highlighted, a significant hematoma can accumulate before manifesting as a shock, making it crucial for healthcare providers to be vigilant even when initial signs are subtle [4]. Delayed recognition of internal bleeding can lead to rapid deterioration in the patient’s condition, emphasizing the need for careful monitoring of any postpartum patient presenting with pain, abnormal vital signs, or a palpable pelvic mass. Even in the absence of overt vaginal bleeding, other indicators such as unexplained hypotension, tachycardia, and persistent lower abdominal pain should prompt further investigation [5].

Diagnostic tools such as ultrasound or CT scans play a critical role in identifying concealed hematomas, allowing for timely intervention. Early recognition of PPH enables prompt management strategies, which may include fluid resuscitation, blood transfusions, and surgical interventions such as hematoma evacuation and vessel ligation [6]. In cases where conservative management is sufficient, close monitoring is essential to ensure stability and prevent complications. Maintaining a high index of suspicion for PPH, particularly in cases with subtle or delayed symptoms, is essential for early diagnosis and effective management. Vigilance in monitoring postpartum patients, combined with appropriate diagnostic and therapeutic interventions, can significantly improve outcomes and reduce the risk of severe complications associated with broad ligament hematomas and other causes of PPH [7]. This report aims to shed light on the clinical presentation, diagnosis, and management of this unusual obstetric complication encountered at Saveetha Medical College and Hospital, Thandalam, Chennai.

## Case presentation

A 22-year-old primigravida at 39 weeks gestational age, diagnosed with hypothyroidism, presented to the outpatient department with a history of abdominal pain occurring intermittently over the past three days. The patient reported a normal perception of fetal movements. Upon examination, the patient was afebrile, exhibited no pallor, and had no pedal edema. The cardiovascular examination revealed normal first and second heart sounds, while the respiratory examination showed normal vesicular breath sounds. The abdominal examination indicated a term, relaxed uterus in cephalic presentation, with fetal heart sounds detected at 150 beats per minute. A speculum examination revealed a healthy cervix and vagina, while on digital examination, the cervix was found to be soft, posterior, 2 cm in length, with an os admitting one finger, and the vertex at -3 station. The pelvic assessment confirmed adequacy. All routine investigations, including hemoglobin levels (11 g/dL) and platelet count (200,000/mm^3^), were within normal limits. Ultrasonography revealed normal amniotic fluid index and Doppler studies.

The patient experienced spontaneous progression of labor, leading to the spontaneous rupture of membranes and the delivery of a live female infant weighing 2.830 kg with APGAR scores of 8/10 and 9/10. The delivery was facilitated naturally with a right mediolateral episiotomy, followed by the performance of an AMSTL (active management of the third stage of labor). The placenta and its membrane were delivered intact, and the episiotomy site was meticulously sutured in layers. Approximately one-hour post-delivery, the patient reported intense pain in the episiotomy region, along with a sensation of defecation and perineal discomfort. On examination, vital signs revealed a blood pressure of 90/50 mmHg, a heart rate of 110 beats per minute, and a palpable boggy mass at the 10-11 o'clock position. The rectal mucosa was found to be free.

**Figure 1:**
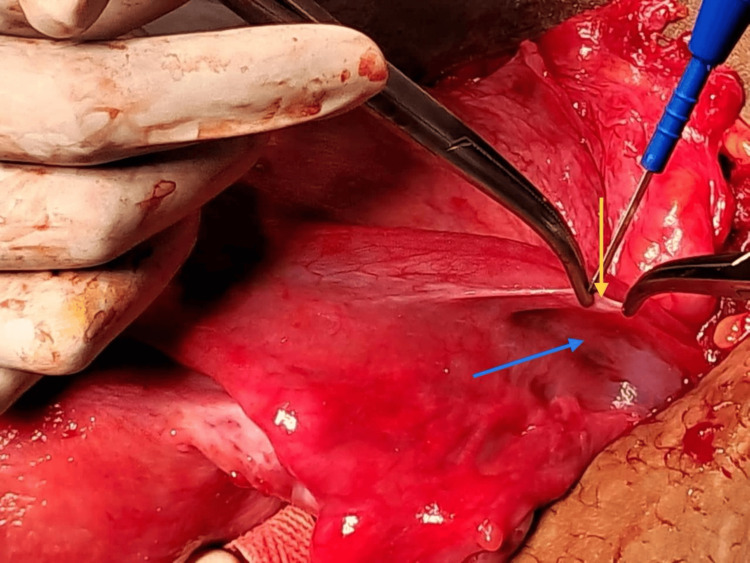
Hematoma located in the right paracolpos supralevator region The yellow arrow shows the opening of the broad ligament; the blue arrow shows the bluish discoloration of the hematoma.

The patient was promptly transferred to the operating theater, where, under general anesthesia, the episiotomy sutures were removed in the mucosal and superficial muscle layers. A hematoma measuring 150 cc, located in the right paracolpos supralevator region, as shown in Figure 1, was evacuated, though the bleeding vessel could not be identified. Additionally, a hematoma was detected in the right anterior paravaginal region and subsequently evacuated. A laparotomy was performed, and the paravesical space hematoma was evacuated after opening the utero-vesical fold. An internal iliac artery ligation was conducted by the vascular surgery team due to the inability to identify the bleeding vessel. The patient experienced a total blood loss of 1200 mL and received 3 units of packed red blood cells and 2 units of fresh frozen plasma. Postoperatively, the patient remained stable. On postoperative day three, the hemoglobin level was measured at 7.4 g/dL, and on postoperative day six, discharge from the wound site was noted. The culture results revealed MRSA, prompting the initiation of treatment with oral linezolid 600 mg twice daily for seven days. The duration of labor was approximately 14-15 hours, and the probable cause of the broad ligament hematoma in this case could be trauma to the blood vessels during childbirth, particularly during the delivery process and the right mediolateral episiotomy.

The patient was followed up at one week and three weeks, during which she exhibited satisfactory progress. The postnatal period was uneventful, with complete healing of the wound and no new complaints. The patient has successfully resumed her normal daily activities after three weeks.

## Discussion

The presented case highlights the rare occurrence of broad ligament hematoma following a routine vaginal delivery, emphasizing the importance of recognizing and managing this uncommon obstetric complication. Despite its rarity, broad ligament hematoma can pose significant challenges due to its potential to cause severe maternal morbidity and mortality if not promptly diagnosed and managed. In this case, the patient's initial presentation with abdominal pain raised suspicion for an underlying obstetric complication, leading to admission for safe confinement. The subsequent development of intense pain over the episiotomy region one-hour post-delivery, coupled with hypotension and tachycardia, signaled a possible PPH-related complication.

Timely intervention was crucial in managing the broad ligament hematoma. The decision to transfer the patient to the operating theater for surgical exploration under general anesthesia was appropriate, given the severity of the symptoms and clinical findings. The evacuation of the supralevator and paravaginal hematomas, along with an internal iliac artery ligation, demonstrates the multidisciplinary approach required for effective management in such cases. The significant blood loss encountered underscores the potential for broad ligament hematoma to lead to substantial maternal morbidity. Adequate resuscitative measures, including blood product transfusions, were essential for maintaining hemodynamic stability and ensuring optimal postoperative recovery.

Furthermore, the identification of MRSA in wound culture highlights the importance of vigilant postoperative monitoring and appropriate antibiotic therapy to prevent infection-related complications. This case shows the need for a high index of suspicion and prompt intervention in managing broad ligament hematoma following vaginal delivery. Clinicians should remain vigilant for signs and symptoms suggestive of this rare complication, particularly in patients presenting with features of PPH. Multidisciplinary collaboration and timely surgical intervention are crucial for achieving favorable outcomes and preventing adverse maternal sequelae.

Varvoutis et al. reported a case where a 22-year-old woman presented with chorioamnionitis and pre-eclampsia post-vaginal delivery, developing symptoms of fever, vomiting, and increased vaginal bleeding. Despite initial treatment and uterine evacuation, she required transfer for further management, ultimately undergoing a hysterectomy due to an infected broad ligament hematoma [8]. In comparison, Ma et al. described a 23-year-old female with a broad ligament pregnancy diagnosed intraoperatively during laparoscopic exploration. The patient underwent resection of the lesion and salpingectomy due to intraoperative hemorrhage and was subsequently diagnosed with pelvic congestion syndrome [9]. Ibrar et al. presented a case of a 37-year-old patient with post-normal vaginal delivery who developed symptoms of abdominal distention, vomiting, and constipation and was diagnosed with broad ligament hematoma and pseudo-colonic obstruction. An exploratory laparotomy was performed, and a caecostomy was carried out, resulting in a successful outcome [10].

Maqbool et al. reported a complex placenta percreta involving the broad ligament and urinary bladder in a twin pregnancy. Despite timely hysterectomy and arterial ligation, the patient did not survive due to massive hemorrhage [11]. Tokuda et al. discussed a case of uterine perforation post-dilation and curettage, leading to a hematoma under the broad ligament. Surgical intervention was required due to clinical deterioration, emphasizing careful observation post-procedure [12]. Kurakula et al. presented a case of uterine rupture in an unscarred uterus, resulting in a broad ligament hematoma noticed during tubal ligation. Surgical treatment was initiated upon clinical deterioration, highlighting the importance of vigilance during postpartum procedures [13]. Bankada et al. described a rare case of bilateral broad ligament hematoma in a twin pregnancy, managed with an emergency cesarian section and hematoma evacuation. The patient had a favorable outcome following blood transfusion and postoperative care [14].

## Conclusions

The presented case report underscores the rarity and clinical significance of broad ligament hematoma following vaginal delivery, emphasizing the necessity for a high index of suspicion and prompt intervention in cases of PPH with atypical presentations. Through comparison with similar cases in the literature, it is evident that broad ligament hematoma can arise from various obstetric conditions and may manifest with diverse clinical symptoms, ranging from abdominal discomfort to hypotension and shock. Timely surgical exploration, hematoma evacuation, and arterial ligation are crucial for preventing adverse maternal outcomes, necessitating close multidisciplinary collaboration and vigilant postoperative monitoring to ensure optimal recovery and minimize complications.
